# Aligned Collagen Sponges with Tunable Pore Size for Skeletal Muscle Tissue Regeneration

**DOI:** 10.3390/jfb14110533

**Published:** 2023-10-24

**Authors:** Natalie G. Kozan, Sean Caswell, Milan Patel, Jonathan M. Grasman

**Affiliations:** Department of Biomedical Engineering, New Jersey Institute of Technology, Newark, NJ 07102, USA

**Keywords:** volumetric muscle loss, biomaterial, skeletal muscle, collagen, porosity, scaffold, tissue engineering, pore size, skeletal muscle tissue engineering

## Abstract

Volumetric muscle loss (VML) is a traumatic injury where at least 20% of the mass of a skeletal muscle has been destroyed and functionality is lost. The standard treatment for VML, autologous tissue transfer, is limited as approximately 1 in 10 grafts fail because of necrosis or infection. Tissue engineering strategies seek to develop scaffolds that can regenerate injured muscles and restore functionality. Many of these scaffolds, however, are limited in their ability to restore muscle functionality because of an inability to promote the alignment of regenerating myofibers. For aligned myofibers to form on a scaffold, myoblasts infiltrate the scaffold and receive topographical cues to direct targeted myofiber growth. We seek to determine the optimal pore size for myoblast infiltration and differentiation. We developed a method of tuning the pore size within collagen scaffolds while inducing longitudinal alignment of these pores. Significantly different pore sizes were generated by adjusting the freezing rate of the scaffolds. Scaffolds frozen at −20 °C contained the largest pores. These scaffolds promoted the greatest level of cell infiltration and orientation in the direction of pore alignment. Further research will be conducted to induce higher levels of myofiber formation, to ultimately create an off-the-shelf treatment for VML injuries.

## 1. Introduction

Volumetric muscle loss (VML) is a condition in which skeletal muscle mass is lost due to a traumatic event, which results in permanent functional impairment of the muscle [1,2]. These injuries may be caused by traumatic events such as sports injuries, surgical procedures, automobile accidents, or combat wounds [3]. Studies that evaluated thousands of battlefield injuries involving US and UK service members who had been injured and evacuated between 2001 and 2014 showed that over 75% of these service members sustained musculoskeletal injuries, and VML was implicated in almost all of these cases [4]. The current standard of treatment for VML is an autologous tissue graft, where a separate, healthy muscle from the patient is removed and implanted into the injury site. This treatment not only involves two surgeries (e.g., tissue harvest and tissue integration into the injured location), but is limited by donor site morbidity, formation of scar tissue, and lack of functional recovery [3,5]. Therefore, there is a clear need to develop alternative approaches to the treatment of VML.

There are varying tissue-engineered approaches that can incorporate growth factors and/or cells within a biomaterial scaffold. Cell-based approaches, namely the addition of cells into an extracellular matrix (ECM)-based scaffold, are advantageous because they enhance muscle regeneration in part by supplying additional myoblasts or satellite cells, the local progenitor cells of skeletal muscle, that can integrate into the host tissue [6]. These treatments are limited, however, due to the high cost of treatment, risks associated with the collection, isolation, and/or expansion of autologous cells, and regulatory hurdles that must be overcome to use these cell therapies in clinical settings [5]. Acellular biomaterial scaffolds have shown promise for use in skeletal muscle regeneration because they provide biophysical and biochemical cues to the injured muscle and thus facilitate the formation of functional myofibers [3]. Such scaffolds can be designed with aligned architecture to match the architectural template of muscle anatomy. These structural cues induce the alignment of myofibers, which is essential for proper force conduction. However, acellular approaches are limited by relatively low amounts of satellite cell migration into the implant site [3]. There is thus a need to improve upon the regenerative capabilities of biomaterial scaffolds used for skeletal muscle tissue engineering to enhance satellite cell recruitment and migration into materials to facilitate further myofiber maturation and alignment.

Biomaterial sponges have been tested for various uses in the field of skeletal muscle tissue regeneration. These 3-dimensional (3D) structures mimic the native 3D architecture of the ECM. Collagen type I is a major component of the local ECM of skeletal muscle and provides structural integrity of the tissue [2]. Previous work has shown that collagen scaffolds support myoblast infiltration and differentiation into myofibers [7], as well as revascularization of damaged tissue [8]. In one study, implanted collagen sponges rapidly induced platelet aggregation, limiting the infiltration of inflammatory cells around the biomaterial and thus resulting in a minimal immune response [7]. The success of collagen in regenerating skeletal muscle is somewhat limited, however, when the collagen is in the form of a hydrogel [9] or a nonaligned scaffold [10].

Research suggests that myofiber formation is enhanced upon aligned scaffolds as opposed to randomly oriented scaffolds [11,12,13]. Alignment of myofibers is necessary for muscle functionality—namely contraction and force transmission, and more closely mimics the native architecture of skeletal muscle tissue [14,15,16]. Various methods of fabricating anisotropic biomaterial scaffolds have been developed, including electrospinning [16,17,18,19,20], microfluidics [20,21,22,23], extrusion bioprinting [20,23,24,25,26,27], stretching [20,28,29,30,31], and freeze drying [20,25,32,33,34]. Unidirectional freeze-drying is a straightforward and cost-effective method of inducing alignment that has been used to create aligned collagen sponges [25]. To develop anisotropic pores, we will apply a temperature gradient to the sponge as it is being frozen [14,35,36], with the hypothesis that aligned pores will facilitate the ingrowth of myoblasts into the bulk of the sponge.

Pore size has an important effect on both the architecture of the scaffold and the behavior of the cells seeded upon the scaffold. The size of pores is critical, not only are they important for mass/nutrient transfer, but they also need to be a size that is appropriate for myoblasts to infiltrate, and subsequently fuse to form myofibers. Many studies have sought to determine the effect of pore size on a variety of cell types, but information on how pore size specifically affects myoblasts is limited. It has been shown that relatively smaller pore sizes (~100 µm as opposed to ~250 µm) can improve the adhesion of cells to both the matrix and neighboring cells, while potentially restricting cell infiltration within the scaffold [33]. Smaller pore sizes have been shown to promote higher density of osteoblasts within collagen-GAG scaffolds (pore size ~96 µm) [37] and of endothelial cells seeded on poly (lactic-co-glycolic) acid (PLGA) films (pore size ~5–20 µm) [38]. In contrast, smaller pore sizes (~38 µm) decrease the viability of other cell populations such as smooth muscle cells and dermal fibroblasts [39]. Pores in the range of 370–400 µm have been shown to direct adipose stem cells to undergo chondrogenesis [40]. Little data exist, however, characterizing the optimal pore size for the growth and proliferation of skeletal muscle myoblasts. Some research suggests that pore sizes around 253 µm improve myoblast infiltration, while pore sizes around 128 µm promote differentiation, as indicated by higher levels of myosin heavy chain (MHC) expression [34]. We plan to create a wide range of pore sizes in collagen sponges and examine their effect on myoblast behaviors, such as proliferation and differentiation. We selected collagen as the bulk material of our scaffolds because of its wide use as a biomaterial and because it has previously been shown to enhance skeletal muscle tissue regeneration. Our goal is to determine an optimal pore size and thus an optimal fabrication method for creating scaffolds optimized for skeletal muscle regeneration.

The goal of this study is to determine the optimal pore size to enhance myoblast proliferation and differentiation. We have developed a custom-made freezing process where we will vary pore size and formation by altering the temperature gradient during the freezing process. This creates the alignment of pores within the structure by allowing the material to freeze unidirectionally from the bottom to the top [41]. We will characterize pore size as a function of freezing rate and determine the ability of myoblasts to infiltrate within sponges to mimic conditions found in vivo during the repair of VML injury. Through these studies, we will establish a structure/function relationship between freezing rate and pore size and determine optimal processing conditions for collagen sponges to be effective in the regeneration of skeletal muscle tissue. Optimizing these characteristics will be a valuable first step in ultimately designing an effective, off-the-shelf treatment for volumetric muscle loss.

## 2. Materials and Methods

### 2.1. Preparation of Collagen Hydrogels

Type I rat tail collagen (Corning, Bedford, MA, USA, CB354249) was combined with 1 N sodium hydroxide and 10X phosphate-buffered saline (PBS; Fisher Scientific, Atlanta, GA, USA) per manufacturer’s instruction to a final concentration of 5 mg/mL collagen (0.5% *w*/*v*) using 1X PBS as the diluent. The 5 mg/mL collagen solution was added to cylindrical polytetrafluoroethylene (PTFE) molds (outer diameter: 3/4 inch; inner diameter: 3/8 inch; McMaster-Carr, Elmhurst, IL, USA), with each mold containing 550 µL of the collagen solution. The molds were placed into a 12-well plate (Fisher Scientific) and incubated at 37 °C for 1 h to allow for biopolymer polymerization and subsequently removed from the incubator, placed into beakers on a shaker plate, and dialyzed against deionized water for 2 h, changing the water every 30 min, to remove excess salts.

### 2.2. Freezing Collagen Gels to Generate Anisotropic Pores

Collagen gels were frozen using an in-house custom-made freezing device to set up a controlled temperature gradient along the samples. To achieve this, PTFE molds were removed from the beakers, returned to 12-well plates, and covered with a rectangular PTFE plate to control the direction of the thermal gradient. The 12-well plate was then placed on top of a pre-chilled aluminum block (−20 °C, −40 °C, −60 °C, −80 °C, or −196 °C) until frozen.

### 2.3. Cooling Aluminum Block to Control Freezing Rate

In all cases, the aluminum block was equilibrated at the indicated temperature for at least 30 min prior to incorporation of the 12-well plate and the entire setup was incubated in the same conditions until the samples were fully frozen. Fully frozen samples were stored at −20 °C until lyophilization. Freezing temperatures of −20 °C or −80 °C were achieved with incubation in appropriately cooled freezers. The freezing temperature of −196 °C was achieved by immersing the aluminum block in liquid nitrogen. The freezing temperatures of −40 °C and −60 °C were achieved via a combination of dry ice, absolute ethanol (Fisher Scientific, Atlanta, GA, USA), and ethylene glycol (Sigma–Aldrich, Burlington, MA, USA), based on previous methods [42]. To achieve a freezing temperature of −40 °C, a 60:40 (*v/v*) solution of ethylene glycol:ethanol was poured on top of dry ice to create a slurry. A 40:60 (*v/v*) solution of ethylene glycol:ethanol was prepared to freeze the samples at −60 °C. The aluminum block was added to either of these mixtures and left until the block reached the appropriate temperature, taking care that the block was not in contact with any pieces of dry ice. The samples were then placed on top of the block and the whole setup was kept in a closed Styrofoam container until the samples were fully frozen.

### 2.4. Lyophilization of Porous Scaffolds

Fully frozen samples were transferred to a lyophilizer (Labconco, Kansas City, MO, USA) to remove the ice crystals. Samples remained in the lyophilizer for 2–3 days, until they were fully dried, after which the sponges were removed from PTFE molds and stored at room temperature until future use.

### 2.5. Pore Size Analysis

To analyze sponge pore size, we conducted a pore size analysis based on the cryosections of our samples, as conducted in previous literature [43,44]. Sponges were incubated in a 30% (*w*/*v*) sucrose solution overnight, and then embedded in optimal cutting temperature (OCT) positioned to cut perpendicular to the pore alignment. The samples were incubated in a vacuum chamber for 2 h to facilitate OCT infiltration into the porous structure, and subsequently frozen. The samples were cryosectioned to create 20 µm thick sections, adhered to microscope slides, and left at room temperature overnight until fully dried.

To visualize the porous structure of the sponges, sliced sections were stained with aniline blue. Tissue sections were washed in deionized water for 3 min to remove excess OCT and submerged in a Coplin jar containing 2.5% aniline blue in 2% acetic acid (Sigma–Aldrich) solution for 2 min. Slides were transferred into a clean Coplin jar and rinsed in running tap water for 2 min. The jar was inverted to remove the water, and two more 2-min rinses were conducted with running water, for a total of 6 min of rinsing. Next, the slides were incubated in 1% (*v/v*) acetic acid for 10 min and rinsed in running water for 6 min (3 rinses for 2 min each), and cover-slipped prior to imaging.

**Figure 1:**
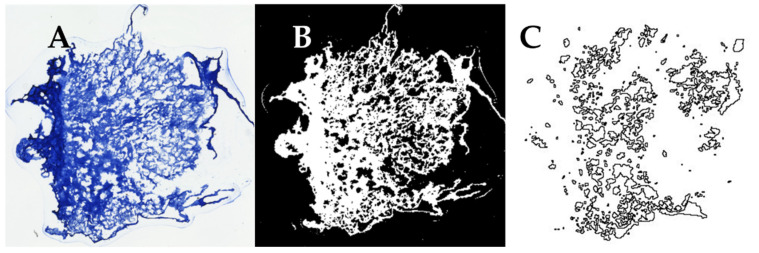
Process of quantifying pore in histological sections of collagen sponges. (**A**) A color image of sponges stained with aniline blue is (**B**) converted to an 8-bit image and thresholded in ImageJ. (**C**) The “Analyze Particles” function in ImageJ was used to outline each pore and used to calculate its diameter.

The samples were imaged using a Keyence BZ-X810 (Keyence Corporation of America, Itasca, IL, USA) fluorescent microscope using a 4X objective, and then ImageJ (Version 1.53t, NIH, Bethesda, MA, USA) was used to conduct a pore size analysis of the images obtained. The images were uploaded into ImageJ (Figure 1A), thresholded (Figure 1B), and the “Analyze Particles” function was used to obtain values for the area of each particle (pore) within the image (Figure 1C). Each area measurement was converted into diameter measurements assuming a circular pore shape. The threshold value was selected by comparing the relative pore size and size of sectioning artifacts across all samples prior to analysis and was consistently applied to each tissue section. The average pore diameters from each scaffold section were then averaged together to obtain the overall average pore diameter for each freezing temperature group.

### 2.6. Scanning Electron Microscopy

Scanning electron microscopy (SEM) was used to examine the architecture of the fabricated scaffolds. After lyophilization, samples were snap-frozen via immersion in liquid nitrogen either perpendicular to or along, the pore alignment. Samples underwent gold/palladium sputter-coating before being imaged. Sputter-coated samples were placed into the chamber of a JEOL JSM-7900F (JEOL USA, Peabody, MA, USA). Samples were imaged at 150× using an acceleration voltage of 2 kV, emission current of 75 µA, and working distance of 10 mm. To confirm the pore analysis conducted with cryosectioning, SEM images were analyzed in ImageJ to measure the pore diameter from each freezing temperature group.

### 2.7. Mechanical Testing

The mechanical properties of collagen sponges were tested using a uniaxial testing machine (Instron, Norwood, MA, USA). Sponges fabricated at each freezing temperature were tested to determine whether temperature has an influence on the strength of the scaffolds. Sponges were placed into the grips of the uniaxial testing machine, where one grip was fixed and the other was attached to an actuator and a 100 N load cell. Sponges were loaded until failure at a rate of 5 mm/min. Load and displacement values were continuously recorded until the end of the test. The point of failure of each sample was identified as the point at which the load dropped to less than 50% of the previous value. Stress was calculated by dividing the force by the initial cross-sectional area of the sponge. A custom script was written in MATLAB 2021a (MathWorks, Natick, MA, USA) and was used to calculate Young’s modulus, ultimate tensile strength (UTS), peak load, and strain at failure for each sample based on the raw data of the stress/strain curve. Using MATLAB, the initial linear portion of the stress vs. strain curve was identified and used to calculate Young’s modulus. The UTS and peak load were determined by finding the maximum stress value in each data set. The strain at failure and peak load values was defined as the point at which the load dropped to less than 50% of its previous value.

### 2.8. Cell Culture

C2C12 myoblasts (CRL-1772, ATCC, Manassas, VA, USA) were routinely subpassaged at 70–80% confluency using standard procedures prior to use in experiments. Collagen sponges from each experimental group were cut to be 3 mm thick and sterilized by immersion in 70% ethanol for 2.5 h. After sterilization, the sponges were rinsed with 1X PBS six times, for five minutes each. DMEM/F12 (Gibco, Waltham, MA, USA) supplemented with 10% fetal bovine serum (Gibco) and 1% antibiotic-antimycotic (Gibco), referred to as myoblast growth medium, was added to each sponge within a low-adherence 24-well culture plate (Fisher Scientific) and were incubated at 37 °C and 5% CO_2_ overnight. After this, the growth medium was removed and C2C12 myoblasts were seeded onto the surface of each sponge at a concentration of 1 million cells/cm^2^. After a one-hour incubation at 37 °C, the sponges were flipped within the well and the other surface was seeded with the same concentration of myoblasts. Once each side of the scaffold was incubated for one hour, sponges were transferred to a new well plate, flooded with medium, and returned to the incubator. After three days, the growth medium was removed and replaced with differentiation medium (DMEM/F12 supplemented with 2% heat-inactivated horse serum (Gibco), 1% insulin-transferrin-selenium (Gibco), and 1% antibiotic-antimycotic, and the medium was replaced every other day.

### 2.9. Cell Metabolism Assay

Cell metabolism was assessed on days 1, 3, and 10 using a cell counting kit-8 (CCK8) assay (Sigma–Aldrich), as per the manufacturer’s instructions. At each indicated day after seeding, the medium was replaced with CCK8 reagent that was diluted in the appropriate culture medium at a ratio of 1:10 and incubated for 1 h at 37 °C and 5% CO_2_. CCK8 solution was also added to acellular collagen sponges as a control. After an hour, 100 µL of the CCK8 solution was removed from each well and pipetted into a 96-well plate. The rest of the CCK8 solution was removed from the sponges and replaced with the appropriate culture medium. The 96-well plate containing the CCK8 solution was placed into a microplate reader (Molecular Devices, San Jose, CA, USA) and read at 450 nm to obtain absorbance values. Absorbance values from acellular controls were subtracted from the experimental groups to verify that increases in absorbance were due to the presence of cells. Absorbance values were then compared between experimental groups or between days to determine whether pore size affects cell growth and viability.

### 2.10. Immunohistochemistry

After ten days in culture, sponges from each treatment group were fixed, cryosectioned, immunostained for myosin, and counterstained with phalloidin and DAPI. Sponges were fixed in 4% (*w*/*v*) paraformaldehyde for 2 h and rinsed four times in 1X PBS for five minutes each. The PBS was replaced with 30% (*w*/*v*) sucrose solution and samples were incubated at 4 °C overnight. The samples were then transferred to plastic specimen molds containing OCT such that sections would be obtained parallel to pore alignment. Samples were placed into a vacuum chamber for 2 h to allow for OCT infiltration and subsequently frozen at −80 °C for at least one hour. The samples were cryosectioned to obtain 10 µm thick sections, adhered to microscope slides, and left at room temperature overnight until fully dried.

Sections were washed in 0.05% (*v*/*v*) PBS-Tween (PBST) for 20 min to remove excess OCT. Samples were blocked with 5% (*w*/*v*) bovine serum albumin (BSA) in PBST for 30 min. The primary solution consisting of antibodies targeted myosin heavy chain (MF 20-s; 1:10; Hybridoma Bank, Iowa City, IA, USA) in 1% (*w*/*v*) BSA/PBST was added to the sections and incubated at room temperature for 2 h. Sections were then washed with PBST three times for 10 min each. Secondary antibody (Alexa Fluor 594; 1:250; Invitrogen, Waltham, MA, USA) and phalloidin (1:500; Alexa Fluor 488; Invitrogen) were diluted in 1% (*w/v*) BSA/PBST, added to the samples, and incubated at room temperature for 1 h. Afterward, the slides were washed in PBST three times for 10 min each. A mounting medium containing DAPI (Abcam, Waltham, MA, USA) was added to the samples, and they were then sealed with coverslips. Once immunostaining was complete, the slides were imaged using the Keyence BZ-X810 fluorescent microscope.

### 2.11. Quantification of Cell Infiltration and Orientation

Immunostained images of cell-laden scaffold sections were analyzed using ImageJ to quantify cell infiltration into the scaffolds, cell density, and orientation of the cells within the scaffold. To measure cell infiltration, the number of cells inside a scaffold was calculated as a percentage of the total number of cells in the image. To do this, the image was initially rotated so that the bottom edge of the scaffold was aligned with the horizontal plane of the image. The “Analyze Particles” function was used to determine the number of DAPI-positive nuclei for both the overall image and the selected inner portion of the scaffold. To measure cell density, the cell count was normalized to the area of the scaffold section. To measure cell orientation, the angle of each nucleus was measured. This was also measured using the “Analyze Particles” function in ImageJ, through which each cell nucleus was considered a particle, and the angle of each particle was reported as an output using this ImageJ function. The angle of pore alignment was also measured in each section by drawing a line in the direction of each pore using brightfield images of the scaffold slices to measure its angle with respect to the horizontal axis. The angles of all cell nuclei and pores in each image were separately averaged, and these values were averaged across experimental conditions to obtain the reported values. The average pore angle was compared to the average cell angle of each experimental condition to determine the degree to which the cells were aligned in the same direction as scaffold pores. At least three sections distributed throughout each sponge were analyzed, with at least four sponges analyzed per freezing temperature group.

### 2.12. Statistics

Data are presented as mean ± standard deviation except where indicated. Statistically significant differences were evaluated using one-way analysis of variance (ANOVA) with Tukey’s post-hoc analysis using the SPSS statistical software (Version 27, IBM^®^, Armonk, NY, USA). For all analyses, differences between conditions were considered significant at *p* < 0.05. Where indicated, a Student’s *t*-test was performed, with *p* < 0.05 indicating significant differences between sample groups.

## 3. Results

### 3.1. Decreasing Freezing Temperature Decreases Pore Size in Collagen Sponges

**Figure 2:**
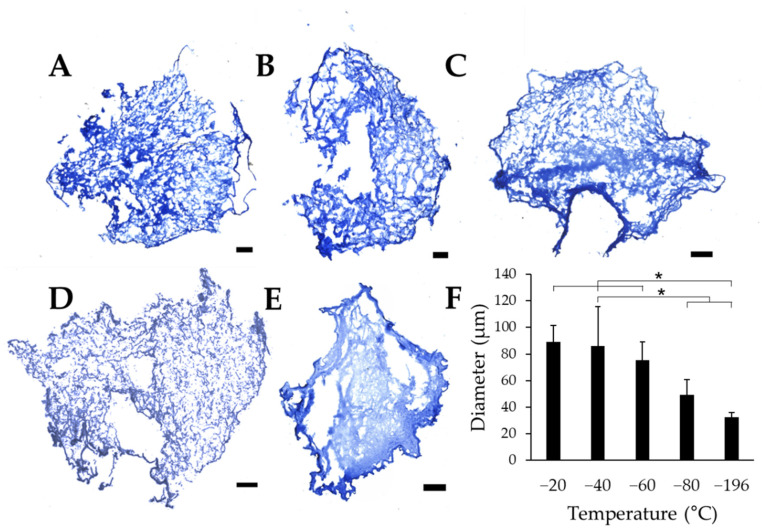
Pore diameters of collagen sponges. Collagen sponges frozen at (**A**) −20 °C, (**B**) −40 °C, (**C**) −60 °C, (**D**) −80 °C, and (**E**) −196 °C. (**F**) Quantification of the average pore diameter of sponges at each freezing temperature. Scale: 500 µm; * indicates significance between indicated groups as determined by one-way ANOVA with Tukey post hoc analysis (*p* < 0.05).

To quantitatively evaluate the effects of freezing temperature on pore size, collagen sponges were frozen on pre-chilled aluminum blocks. After lyophilization, cross-sectional slices of the samples were stained with aniline blue to quantify pore size using ImageJ. Each scaffold section revealed numerous pores, with qualitative differences in pore size between collagen sponges frozen at −20 °C (Figure 2A), −40 °C (Figure 2B), −60 °C (Figure 2C), which show clear pores between the fibrillar collagen matrix. Conversely, sponges frozen at −80 °C (Figure 2D), and −196 °C (Figure 2E) reveal pores that appear much smaller than those in warmer freezing conditions. Overall, sponges frozen at −20 °C, −40 °C, and −60 °C appear to have larger pores than sponges frozen at −80 °C and −196 °C. Quantification of pore diameter revealed that changing the freezing temperature of the collagen sponges significantly affects the pore size within each freezing condition. We found that pore diameter significantly decreases with decreasing freezing temperature (Figure 2F). Interestingly, sponges frozen at −20 °C, −40 °C, and −60 °C all had similar pore sizes, which were collectively significantly larger than those in scaffolds frozen at lower temperatures (−80 °C and −196 °C). The warmer freezing temperatures (−20 °C, −40 °C, and −60 °C) resulted in approximately the same pore size range (75.5–88.9 μm), and the two colder temperatures analyzed (−80 °C and −196 °C) generated distinct pore sizes despite the lack of statistical significance between these experimental conditions.

### 3.2. SEM Reveals Alignment and Interconnectivity of Pores

**Figure 3:**
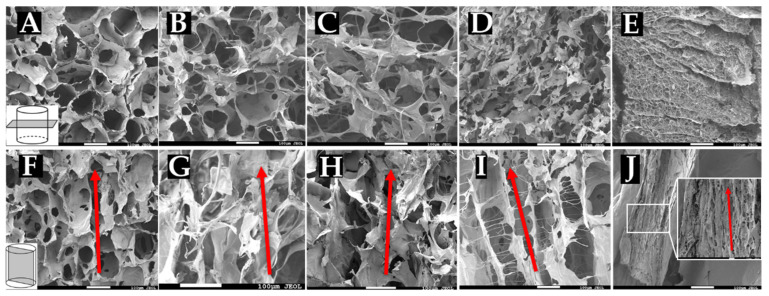
Scanning electron microscopy images of collagen sponges. Collagen sponges frozen at (**A**,**F**) −20 °C, (**B**,**G**) −40 °C, (**C**,**H**) −60 °C, (**D**,**I**) −80 °C, and (**E**,**J**) −196 °C. Cross sections of the sponges (**A**-**E**) reveal interconnected porous structure, while longitudinal cuts of the sponges (**F**–**J**) reveal longitudinal alignment of pores. Red arrows indicate direction of alignment. Scale: 100 µm.

To confirm pore measurements obtained from cryosectioning and that pores are indeed longitudinally aligned with the scaffold, sponges were snap-frozen and prepared for SEM imaging. Collagen sponges fabricated at −20 °C (Figure 3A,F), −40 °C (Figure 3B,G), −60 °C (Figure 3C,H), −80 °C (Figure 3D,I), and −196 °C (Figure 3E,J) were cut either cross-sectionally or longitudinally and imaged using SEM. Cross-sectional images (Figure 3A-E) reveal interconnected porous structures within the sponges. Quantification of pore size from these images resulted in similar values as those found in our analysis of cryosections (data not shown). These images thus confirm our pore measurements, where sponges frozen at warmer temperatures (−20 °C, −40 °C, and −60 °C) all appear to have similarly sized pores evenly dispersed throughout the matrix. Sponges frozen at colder temperatures had markedly smaller pore structures, with −196 °C sponges showing minute pores that were not readily observable with our cryosectioning method. Sponges frozen at −20 °C, −40 °C, and −60 °C seemed to contain thin collagen walls surrounding the pores, while the arrangement of the smaller pores appeared to interrupt these structures in the sponges frozen at −80 °C and −196 °C. Longitudinal images (Figure 3F–J) reveal the longitudinal alignment of pores throughout the length of the sponges. These images also revealed fibrillar structures within the sponges formed at lower temperatures, particularly −196 °C, which showed a densely packed matrix with small pore features. Sponges frozen at −80 °C and −196 °C appear to contain more lamellar or channel-like structures formed by the alignment of pores in a single direction. Sponges frozen at −20 °C, −40 °C, and −60 °C appear to have less of a lamellar architecture but still maintain a clear alignment of pores throughout the scaffold.

### 3.3. Pore Size Affects Stiffness but No Other Mechanical Properties of Collagen Sponges

**Figure 4:**
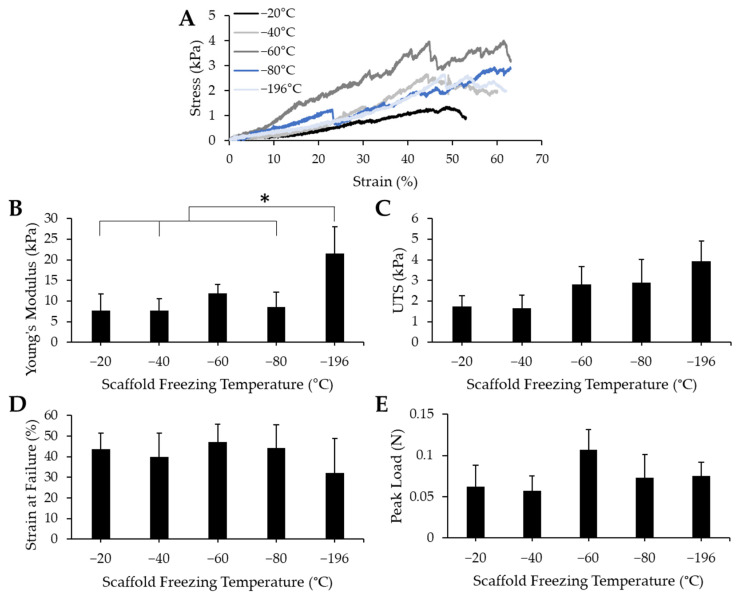
Mechanical properties of collagen sponges fabricated at various freezing temperatures. (**A**) Representative stress/strain curves for each sample reveal similar points of failure. Quantification of (**B**) Young’s modulus show a significant difference between −196 °C and −20 °C, −40 °C, and −80 °C, while measurement of (**C**) ultimate tensile strength (UTS) (**D**), strain at failure and (**E**) load at failure reveals a lack of significant differences between values for each freezing temperature group. * indicates significance between indicated groups as determined by one-way ANOVA with Tukey post hox analysis (*p* < 0.05). Error bars represent standard error of the mean.

To determine whether pore size impacted the mechanical properties of the bulk collagen sponges, we conducted uniaxial tensile testing to measure Young’s modulus, ultimate tensile stress (UTS), strain at failure, and peak load. Collagen sponges fabricated by freezing at −20 °C, −40 °C, −60 °C, −80 °C, and −196 °C were mounted onto a uniaxial Instron and were pulled until failure. Representative stress/strain curves (Figure 4A) reveal that failure of the materials occurred at similar strain values. The toe region, or the portion of the curve that represents the uncrimping of collagen fibrils, manifested similarly among all the freezing groups—they show a period of increased elongation with only a slight increase in stress. While the toe region of scaffolds frozen at −60 °C appeared to be slightly shorter, this did not impact the overall mechanical properties. The linear regions of these stress/strain curves contain several oscillations, however, this seems to be consistent throughout all freezing groups and likely is attributable to micro tears between pores that may have occurred throughout the testing process. The Young’s modulus (Figure 4B), UTS (Figure 4C), strain at failure (Figure 4D), and peak load (Figure 4E) were all determined from the stress/strain curves. There were minimal differences between each treatment condition, except in the Young’s modulus values, where the Young’s modulus of the −196 °C sponges was significantly higher than that of the −20 °C, −40 °C, and −80 °C sponges. From these data, we can infer that pore size does not affect the UTS, strain at failure, or peak load of collagen sponges. Pore size does affect the Young’s modulus: the smallest pore size generated induced a stiffening effect which resulted in the highest average Young’s modulus value.

### 3.4. Collagen Pore Size Does Not Impact Myoblast Proliferation

**Figure 5:**
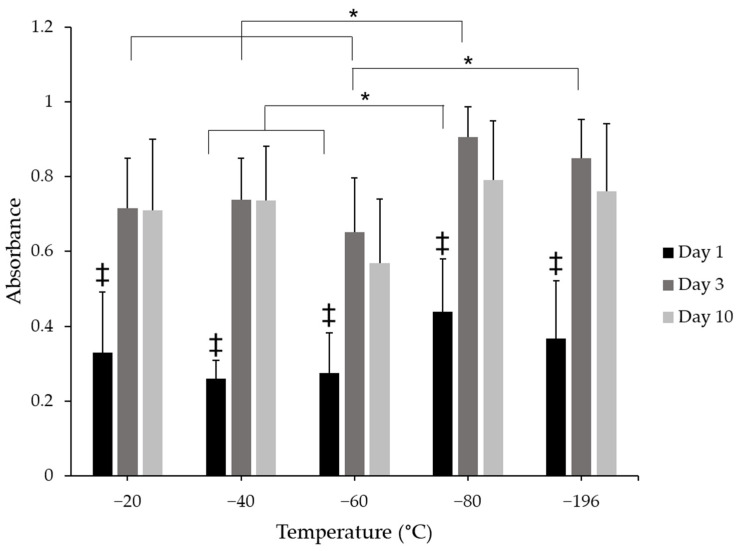
CCK8 metabolic analysis of C2C12 myoblasts cultured on collagen sponges. Absorbance values show increases in cell metabolism levels between day 1 and days 3 and 10. * indicates significance between indicated groups across treatment conditions at the same time point and ‡ indicates significance between day 1 and days 3 and 10 within the same freezing temperature as determined by one-way ANOVA with Tukey post hox analysis (*p* < 0.05). Error bars represent standard error of the mean.

To evaluate the effects of pore size on cell proliferation, cell number was indirectly assessed through the use of the CCK8 metabolic assay. There were changes in cell metabolism between days 1, 3, and 10 (Figure 5). Cell metabolism significantly increased from day 1 to days 3 and 10 in all scaffold groups. There were no qualitative differences between days 3 and 10 for any freezing group, suggesting no further increase in proliferation between these time points, which was expected given that the sponges were cultured in a differentiation medium during this time. Sponges frozen at −80 °C supported higher cell metabolism levels than −40 °C and −60 °C scaffold groups on day 1, suggesting that more cells were initially present on the −80 °C scaffolds. On day 3, cell metabolism from the −80 °C group was significantly greater than −20 °C, −40 °C, and −60 °C experimental groups, which may have resulted from a higher initial seeding density, however, these differences did not persist at 10 days in culture for any treatment group; there were no significant differences between any condition at this time point. These data suggest that metabolism, and indeed cell number, may have reached a saturation point between days 3 and 10 in culture and could also suggest that myoblasts were differentiating rather than proliferating.

### 3.5. Pore size Influences Myoblast Infiltration and Orientation

**Figure 6:**
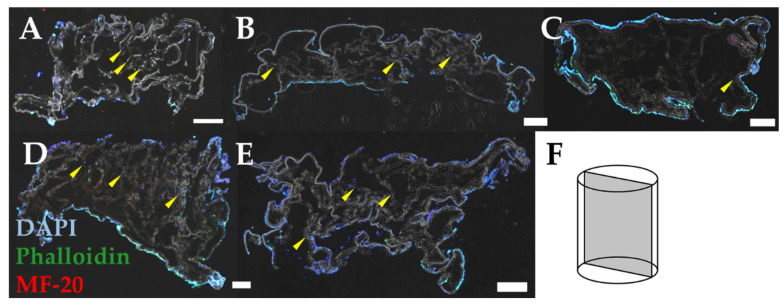
Stained images of myoblasts in collagen sponges. Immunostaining reveals myoblasts seeded on scaffolds fabricated at (**A**) −20 °C, (**B**) −40 °C, (**C**) −60 °C, (**D**) −80 °C, and (**E**) −196 °C. Images represent a (**F**) longitudinal section of each scaffold. Yellow arrowheads indicate representative cells which have infiltrated the scaffolds. Results suggest some qualitative differences in cell infiltration, specifically that less infiltration is occurring in the −60 °C scaffold. Scale: 500 µm.

To determine the effect of pore size on myoblast infiltration and orientation, we cultured myoblasts on sponges for 10 days, immunostained them to visualize cell shape, and loaded these images into ImageJ for analysis. Immunostaining of longitudinal sections of the sponges revealed the infiltration of myoblasts into sponges frozen at −20 °C (Figure 6A), −40 °C (Figure 6B), −60 °C (Figure 6C), −80 °C (Figure 6D), and −196 °C (Figure 6E). Each section was positioned to visualize a longitudinal view of the cell-laden sponges, where pores should be positioned along the vertical axis of the image (Figure 6F). DAPI-positive staining shows myoblast infiltration into each scaffold. Some scaffold groups (−60 °C and −80 °C) featured more myoblasts on the surface of the scaffolds, with little to no myoblasts within the porous structure of the scaffolds. Other groups, such as −20 °C showed infiltration of numerous myoblasts throughout the scaffold structure (yellow arrowheads). In all scaffold groups, however, there were fewer cells that had infiltrated the scaffold compared to the number of cells that remained on the surface of the scaffold. Nonetheless, each scaffold condition did support cell infiltration.

**Figure 7:**
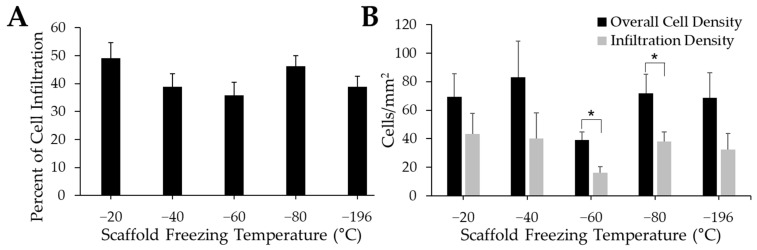
Influence of pore size on cell infiltration. Quantification of cell infiltration indicates a slight increase in infiltration (**A**) in −20 °C sponges. Cell density quantification (**B**) reveals a significantly lower density of cells within the −60 °C and −80 °C when compared to the overall cell density. * indicates significance between groups as determined by Student’s *t*-test (*p* < 0.05).

The percentage of infiltrating myoblasts indicates how many myoblasts have moved from the surface of the scaffold into its interior porous structure (Figure 7A). Qualitatively, the −20 °C group had the highest level of cell infiltration. While the −40 °C, −60 °C, and −196 °C groups had qualitatively lower percentages of myoblast infiltration compared to scaffolds frozen at −20 °C and −80 °C, there were no significant differences between experimental groups. The difference between the overall cell density quantified in each tissue section and the cell density quantified within the scaffold (“infiltration density”) was also measured to corroborate our cell infiltration data (Figure 7B). While the overall cell density was higher than the infiltration density for all groups, there were no significant differences between treatment groups. However, there were qualitatively lower cell densities on the −60 °C scaffolds compared to all other experimental groups. The overall cell density was significantly higher than the infiltration density for the −60 °C and −80 °C groups, while there were no differences between overall cell density and infiltration density for any other experimental group.

**Figure 8:**
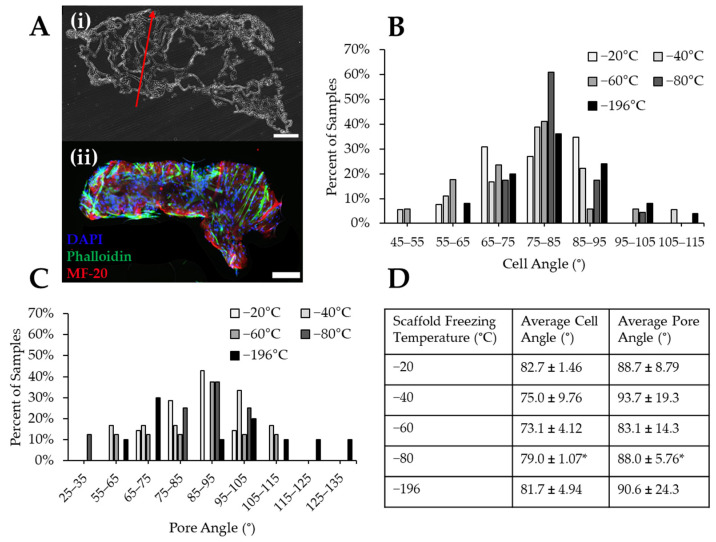
Cell and pore orientation within collagen sponges frozen at different temperatures. (**A**) (**i**) Representative histological section of a collagen sponge imaged with brightfield to illustrate the angle of pore alignment (red arrow). (**ii**) Immunostaining reveals cell alignment within a longitudinal section of a cell-seeded −20 °C collagen sponge. Scale: 500 µm. Quantification of the binned angles of (**B**) cell nuclei and (**C**) pores. (**D**) Comparison of the cell angle to pore angle of each scaffold freezing temperature reveals that the angle of cell orientation is similar to the pore orientation, except for the −80 °C group. * indicates significance between average cell and pore angles within the same temperature condition as determined by Student’s *t*-test (*p* < 0.05).

To determine the impact of pore size on myoblast alignment, pore and cell nucleus angle were measured with ImageJ. In some groups such as scaffolds frozen at −20 °C, immature myofibers were found to form through the pores in the collagen scaffolds, indicated by the presence of MF-20-positive staining (Figure 8A). The myoblast nuclei angles were measured, aggregated into 10° bins, and plotted as a histogram. Myoblast nuclei tended to align between 75–85° of each image (Figure 8B) with almost all cells aligning within 30° (i.e., 65–95°). The pore angles of each scaffold were also measured, aggregated into 10° bins, and plotted as a histogram (Figure 8C). These data indicated that most scaffolds contained pores oriented at angles between 75–105° (Figure 8C). Collagen sponges frozen at −20 °C and −80 °C produced scaffolds with the most aligned pores, while the other experimental groups had wider distributions of pores throughout the analyzed histogram bins. Myoblasts cultured on scaffolds frozen at −80 °C aligned more uniformly than other scaffold groups, as approximately 60% of cells in −80 °C scaffolds were oriented at an angle between 75–85°. However, the cells in the −80 °C scaffolds did not align in the direction of the pores, which is necessary for the regeneration of unidirectionally aligned myofibers. This is indicated by a significant difference found between the myoblast and scaffold pore angles (Figure 8D). There were no significant differences between pore and cell angle for any other freezing groups, indicating that cells in these scaffolds generally aligned in the direction of pore alignment (Figure 8D). The scaffolds frozen at −20 °C resulted in the tightest distribution of pore alignment in the longitudinal direction and also displayed the highest percentage of pore alignment being found to be 85–95°. Qualitatively, scaffolds frozen at −20 °C also had the smallest differences between the cell and pore angles.

## 4. Discussion

Consideration of biomaterial scaffold architecture is a critical characteristic when seeking to optimize materials for skeletal muscle tissue regeneration. Porosity is not only essential for the transfer of waste and nutrients, but also for cell survival, infiltration, and growth. Current biomaterials have been shown to enhance functional recovery after VML injury. However, recovery is limited by scarring and misaligned materials that lead to the formation of misaligned myofibers, which ultimately limit the ability of the regenerated muscle to produce force [45,46]. Our unidirectional freezing method facilitated the longitudinal alignment of pores within collagen sponges, providing the necessary structure for aligned myofiber formation. The alignment of myoblasts within the porous architecture of the scaffolds demonstrates our ability to fabricate materials to control cell orientation and thus provides a foundation for aligned myofiber formation. In future studies, we will optimize cell seeding conditions and biochemical properties of the collagen matrix to stimulate the formation of a dense, mature myofiber matrix within our scaffolds.

Control of pore size in biomaterial scaffolds has been accomplished via various freezing methods. One commonly used method is placing the sample into a lyophilizer containing a shelf console and varying the final shelf temperature [37,47,48,49,50]. While changing the final temperature of the freeze-dryer shelf can significantly impact the resulting pore size found in scaffolds, this parameter by itself will result in isotropic pore formation [48]. Alternatively, pore size and pore alignment can be controlled simultaneously by varying the rate at which the temperature of the shelf decreases to reach the final shelf temperature. This gradual decrease in temperature results in directional freezing of the scaffold [51]. Directional freezing of biomaterials induces pore anisotropy within the biomaterial, as found in studies where biomaterial scaffolds were placed on a cooled metal block and frozen, thus inducing a thermal gradient across the scaffold [35,41,47,52]. This is also the method we employed in the present study. We sought to develop a system to generate tunable, precise pore sizes within collagen scaffolds while simultaneously inducing anisotropy with directional freezing and subsequent lyophilization. Our method allowed us to achieve these outcomes, and interestingly, we found two separate ranges of pore sizes: freezing temperatures above −60 °C generated similar pore sizes of approximately ~80 µm in diameter and lower freezing conditions produced ~30–50 µm diameter pores. Other studies investigating collagen sponges sourced from various tissue types showed that sponges frozen at −20 °C, −80 °C, and −196 °C contained pores that were 89 ± 24 µm, 42 ± 6 µm, and 24 ± 4 µm, respectively [53]. These numbers align with those generated in our study. While the precise pore size may vary, the results from previous work demonstrate the same trend in pore size: decreasing the freezing temperature of the scaffold decreases the average pore size [47,54]. We thus confirmed that the freezing method we developed produces the desired results and is supported by previous literature.

When designing a scaffold for use in tissue engineering, the mechanical properties of the scaffold should be similar to those of the native tissue being repaired. Scaffold stiffness acts as a mechanical cue to regulate cell behaviors, including adhesion and differentiation [55,56]. We, therefore, sought to determine whether the mechanical properties of our scaffolds were comparable to that of skeletal muscle and whether altering the pore size would impact the strength of the materials. The range of stiffness values of our scaffolds (7.7–21.5 kPa) is similar to the stiffness range of native tissue (12–16 kPa) [14,57]. Myoblasts cultured on either a soft substrate (Young’s modulus of <5 kPa) or a stiff substrate (Young’s modulus of >23 kPa) for two weeks expressed low levels of actin and myosin. Higher levels of actin and myosin expression were found when myoblasts were cultured on substrates with Young’s modulus between 6.5–17 kPa [58]. Myotubes have also been shown to exhibit optimal differentiation on substrates having tissue-like elasticity, (i.e., a stiffness of 12 kPa) [59]. Since the stiffness of our sponges mainly falls within the range listed, our sponges fit the criteria for supporting myofiber growth. While our −196 °C sponges have a Young’s modulus that exceeds that range, we expect that it should still support myofiber growth since this stiffness does not exceed 23 kPa, and because our data show that myoblasts will freely penetrate the porous structure of these scaffolds. The higher Young’s modulus of the −196 °C sponges may be due to the presence of dense areas of collagen between the small pores within these scaffolds, as seen in the SEM images, contributing to the stiffness of the material while not impacting other load-bearing properties. Immature myofibers were observed throughout our scaffolds, and future work will focus on enhancing myofiber maturity. The stiffness values of our collagen sponges are also similar to the reported values of other collagen sponges (10 kPa) [60]. Collectively, our data indicate that these collagen sponges should support the growth and differentiation of myoblasts.

It is interesting to note that while the number of cells in our scaffolds increased between days 1–3, there were no changes between days 3–10. The initial increase in cell number in the first 3 days supports the idea that myoblasts are proliferating, which is expected because they were cultured in a growth medium during this time. The lack of any increase in metabolic activity between days 3–10 of culture suggests that the myoblasts switched from a proliferation phase to a differentiation phase, as cell number is not expected to increase as myoblasts fuse to form myofibers. This was also likely because we transitioned the culture to a differentiation medium at this time point, which has been shown to limit myoblast proliferation while supporting differentiation [61]. The conclusion that differentiation was occurring in these scaffolds is supported by immunostaining images such as that found in Figure 7A, which reveals myofiber formation. This research was conducted to determine whether changing the pore size of the scaffold significantly affects myoblast behavior. To our knowledge, the ideal pore size for growth and differentiation of myoblasts has not been widely studied. Studies have shown that pore size affects certain characteristics of cells such as adhesion [37,49], proliferation [47,49], and infiltration and migration [49,50]. Through our studies, we found that the pore size range of our scaffolds does not seem to greatly affect the proliferation or differentiation of myoblasts.

We determined that changing the pore size affects the ability of myoblasts to infiltrate the scaffold. Our data demonstrate that scaffolds with larger pores, such as scaffolds frozen at −20 °C, facilitate qualitatively more cell infiltration than scaffolds frozen at lower temperatures. These findings are also supported by our observation that significantly fewer cells infiltrated into the porous architecture of scaffolds frozen at −60 °C and −80 °C, despite there being no significant differences between the overall cell density and the infiltration density in these scaffolds. These findings are corroborated by a separate study in which C2C12s were found to have higher levels of proliferation on porous membranes with relatively larger pores (1.5 µm versus 0.5 µm) [62]. Lack of significance between groups for myoblast infiltration could be a result of a variety of reasons including the fact that myoblasts may interact with collagen sponges uniformly regardless of pore size, as all of these pores are greater than or equal to the size of myoblasts. However, our data also suggest that fewer cells were able to move into the −60 °C and −80 °C scaffolds from the surface of the scaffold than cells seeded on scaffolds from the other freezing groups. This may be due to the pores not being large enough; the size of a skeletal muscle cell nucleus is between 20–40 µm, and pores near this size, particularly with the lower freezing temperatures, may be too narrow for cell infiltration and growth. When myoblasts were seeded on scaffolds with a 30 µm gap between fibers and a 50 µm gap between fibers, there were fewer myofibers on the scaffolds with the smaller gap size [63]. Alternatively, there may have been some local variations in cell seeding density resulting from the cell solution sliding off the scaffold. While we used non-treated polystyrene dishes to conduct these experiments to remove the ability of the myoblasts to adhere to the plastic substrate, we cannot exclude this as a possibility. Taken together, the scaffolds fabricated at −20 °C may be ideal for skeletal muscle applications, as more cells were able to infiltrate these scaffolds, and thus more myofibers can potentially form within them.

Previous work by Chung et al. indicated that sponges frozen at lower temperatures (−70 °C and −196 °C) would generate more homogenous pore sizes within scaffolds, while pore sizes generated in scaffolds frozen at higher temperatures (−20 °C) would be more heterogeneous [64]. Based on those data, we would expect our −20 °C freezing condition to have the least pore alignment, and thus the least cell alignment, of all of our experimental conditions. However, our study shows that scaffolds frozen at −20 °C supported qualitatively more pore alignment in the longitudinal direction than scaffolds in other freezing temperature groups, as evidenced by the observation that most of the pores were oriented at an angle between 85–95°. Based on these data, we can infer that the scaffolds fabricated at −20 °C will better facilitate the alignment of myofibers in the longitudinal direction, as indicated by our cell alignment data. This is ideal for use in skeletal muscle regeneration, as we hope to achieve longitudinal alignment of myofibers within an implanted scaffold to allow for a more efficient transmission of uniaxial force. Taken together, our data indicate that scaffolds fabricated at −20 °C have the most potential for enhancing skeletal muscle regeneration. In future studies, we aim to incorporate growth factors to further enhance myoblast infiltration and support the formation of mature myofibers within our scaffolds.

## 5. Conclusions

In this study, we sought to fabricate collagen sponges with tunable pore sizes and determine the ideal pore size for myoblast infiltration, proliferation, and differentiation. We demonstrated that our custom freezing method can be used to generate tunable pore size within collagen scaffolds. We were able to create environments with specific temperatures using readily available freezers, liquid nitrogen, or a combination of dry ice, ethanol, and ethylene glycol: −20 °C, −40 °C, −60 °C, −80 °C, and −196 °C. Varying the temperature in this manner distinctly altered the size of pores found in collagen sponges. We found that our freezing mechanism produced scaffolds with pore diameters in the range of 76–89 µm. Scaffolds fabricated at −20 °C contain pores with a diameter of 88.9 ± 17.6µm, and these scaffolds provide the optimal structure for myoblast behavior. These scaffolds supported the most myoblast infiltration and facilitated the most cell alignment in the direction of the pores. These characteristics are ideal for the formation of myofibers for the efficient transmission of uniaxial force. Our findings on the optimal pore size for myoblast proliferation and differentiation will help us to work toward designing an implantable scaffold for VML treatment, and they can also help to guide any material being used for skeletal muscle regeneration toward increased efficacy.

## Data Availability

The data presented in this study are available in the article. Raw data can be provided upon reasonable request.
